# Role of the AMPK/ACC Signaling Pathway in TRPP2-Mediated Head and Neck Cancer Cell Proliferation

**DOI:** 10.1155/2020/4375075

**Published:** 2020-11-14

**Authors:** Kun Li, Lei Chen, Zhangying Lin, Junwei Zhu, Yang Fang, Juan Du, Bing Shen, Kaile Wu, Yehai Liu

**Affiliations:** ^1^Department of Otorhinolaryngology, Head and Neck Surgery, The First Affiliated Hospital of Anhui Medical University, Hefei, Anhui 230022, China; ^2^Department of Health management center, The First Affiliated Hospital of Anhui Medical University, Hefei, Anhui 230022, China; ^3^Department of Urology, The First Affiliated Hospital of Anhui Medical University, Hefei, Anhui 230022, China; ^4^Institute of Urology, Anhui Medical University, Hefei, Anhui 230022, China; ^5^Department of Physiology, School of Basic Medical Sciences, Anhui Medical University, Hefei, Anhui 230032, China

## Abstract

Transient receptor potential polycystic 2 (TRPP2) exerts vital roles in various types of cancer; however, its underlying mechanisms remain largely unknown. This study is aimed at investigating whether knockdown of TRPP2 affected the AMP-activated protein kinase (AMPK)/acetyl-CoA carboxylase (ACC) signaling pathway and the proliferation of HN-4, cell line originating from human oral and hypopharyngeal squamous cell carcinoma. In addition, the interactions among AMPK/ACC, AMPK/protein kinase RNA-like endoplasmic reticulum kinase (PERK)/eukaryotic initiation factor 2*α* (eIF2*α*) and TRPP2/PERK/eIF2*α* signaling pathways, and their association with cell proliferation were also explored. The results showed that the relative expression levels of phosphorylated (p)-ACC, p-PERK, and p-eIF2*α* in HN-4 cells were significantly increased following treatment with 5-aminoimidazole-4-carboxamide-1-*β*-D-ribofuranoside (AICAR) and significantly decreased in cells treated with compound C. Therefore, consistent with previous studies, the AMPK/ACC and AMPK/PERK/eIF2*α* signaling pathways were upregulated and downregulated following treatment with an AMPK agonist and inhibitor, respectively. Furthermore, TRPP2 knockdown decreased p-PERK and p-eIF2*α* expression levels and increased those of p-AMPK and p-ACC. Additionally, knockdown of TRPP2 increased HN-4 cell proliferation, while treatment with an AMPK inhibitor or agonist increased or inhibited TRPP2-specific siRNA-mediated cell proliferation, respectively. In conclusion, silencing of TRPP2 expression increased HN-4 cell proliferation via inhibiting the PERK/eIF2*α* signaling pathway, while the AMPK/ACC signaling pathway was possibly activated by a feedback mechanism to reduce enhanced cell proliferation.

## 1. Introduction

Head and neck cancer (HNC), originating from the mucosal epithelium of the hypopharynx, nasopharynx, oropharynx, larynx, paranasal sinuses, and nasal and oral cavities, is one of the most common cancer types worldwide [1, 2]. Data from 2018 estimated that there were approximately 840,000 new cases and 430,000 deaths from HNC, accounting for about 4.6% of all cancers [3]. Although current treatments of HNC, including surgical, chemotherapeutic, immunotherapeutic, radiotherapeutic, gene therapeutic, and early detection approaches, have been widely explored, the 5-year overall survival rate remains relatively poor, with 40-60% of cases advancing to uncontrolled invasion and metastasis [4–8]. Increasing evidence has shown that immunotherapy and gene therapy exhibit distinct advantages compared with other types of therapy. However, the response rates to both treatments remain low in some patients due to some limitations that still need to be overcome [7, 8]. Therefore, further studies are required to identify novel therapeutic targets in HNC.

Transient receptor potential polycystic 2 (TRPP2), previously known as polycystin-2 (PKD2 or PC2), is a nonselective cation channel encoded by the *PKD2* gene. TRPP2 is a membrane-associated protein that regulates cell signal transduction and the intracellular calcium (Ca^2+^) concentration [9]. Loss-of-function mutations in TRPP2 leads to autosomal dominant polycystic kidney disease (ADPKD) via promoting cell proliferation and fluid secretion, while tumor necrosis factor-*α*- (TNF-*α*-) mediated suppression of TRPP2 accelerates Hep-2 cell (a cell line originating from human laryngeal squamous cell carcinoma) proliferation [10, 11]. Emerging evidence has demonstrated that TRPP2 regulates the endoplasmic reticulum (ER) Ca^2+^ concentration and serves as an antiapoptotic cation channel localized in the ER membrane [12]. These findings indicate that TRPP2 exerts vital roles in cell apoptosis and proliferation. The regulation of these processes may provide a potential novel target for the treatment of several types of cancer. However, the underlying mechanisms of TRPP2 remain unclear.

AMP-activated protein kinase (AMPK), a kinase consisting of a catalytic *α* and regulatory *β* and *γ* subunits, phosphorylates and inhibits acetyl-CoA carboxylase (ACC) to regulate protein acetylation, fatty acid synthesis and oxidation, cell proliferation, and tumor growth [13–16]. It has been reported that metformin and 5-aminoimidazole-4-carboxamide-1-*β*-4-ribofuranoside (AICAR), two AMPK agonists, inhibit the proliferation of retinoblastoma and prostate cancer cells via the AMPK/ACC and AMPK/mammalian target of rapamycin (mTOR) signaling pathways [16, 17]. Furthermore, the blockade of ACC inhibits prostate cancer cell proliferation via reducing lipid synthesis and increasing fatty acid oxidation, which is similar to inhibitory phosphorylation of ACC by AMPK [18]. Therefore, the AMPK signaling pathways are considered therapeutic targets for HNC.

Protein kinase RNA-like endoplasmic reticulum kinase or pancreatic ER kinase (PERK), an ER transmembrane protein kinase involved in ER stress, has been shown to mediate cell cycle progression by acting as a proximal effector of the mammalian unfolded protein response signaling pathway [19]. PERK acts as the eukaryotic initiation factor 2*α* (eIF2*α*) kinase that phosphorylates eIF2*α* to inhibit protein synthesis and cell growth in ER stress [20, 21]. In addition, AMPK serves as an upstream activator of PERK, while the AMPK/PERK/eIF2*α* signaling pathway plays an important role in protein synthesis and cancer cell proliferation [22–25]. It has been also demonstrated that the TRPP2/PERK/eIF2*α* signaling pathway is involved in the cell proliferation process [26]. However, the interactions among AMPK/ACC, AMPK/PERK/eIF2*α*, and TRPP2/PERK/eIF2*α* signaling pathways in cancer cell proliferation have not been clearly elucidated.

Therefore, the aims of the present study were to explore the interactions among AMPK/ACC, AMPK/PERK/eIF2*α*, and TRPP2/PERK/eIF2*α* signaling pathways and whether TRPP2 knockdown affects the AMPK signaling pathway and proliferation of HN-4 cells, cell line originating from human oral, and hypopharyngeal squamous cell carcinoma. To achieve these goals, it was first established that the functions of the AMPK/ACC and AMPK/PERK/eIF2*α* signaling pathways in HN-4 cells were consistent with those previously demonstrated in other cell lines. The expression levels of ACC, phosphorylated (p)-ACC, PERK, p-PERK, eIF2*α*, and p-eIF2*α* were determined in HN-4 cells pretreated with compound C (an AMPK inhibitor) or AICAR (an AMPK agonist) using western blot analysis. Furthermore, following transfection of HN-4 cells with TRPP2-specific small interfering RNA (siRNA); the changes in AMPK, p-AMPK, ACC, p-ACC, PERK, p-PERK, eIF2*α*, and p-eIF2*α* expression levels; and cell proliferation rates were determined. Finally, to further investigate the effects of TRPP2 silencing in the AMPK/ACC signaling pathway and cell proliferation, HN-4 cells were treated with an AMPK inhibitor (compound C) or agonist (AICAR).

## 2. Materials and Methods

### 2.1. Cell Culture, Transfection, and Reagents

HN-4 cell line was purchased from the American Type Culture Collection (ATCC) and cultured in Dulbecco's modified Eagle medium and Eagle's minimum essential medium (both from Wisent, Inc.) supplemented with 10% fetal bovine serum (FBS; Gibco; Thermo Fisher Scientific, Inc.), 100 kU/l penicillin, and 100 mg/l streptomycin, at 37°C in an incubator containing 5% CO_2_. HN-4 cells were first transfected with TRPP2-specific siRNA (sense, AACCUGUUCUGUGUGGUCAGGUUAUdTdT) (Shanghai GenePharma Co., Ltd.) using Lipofectamine 3000 (Invitrogen; Thermo Fisher Scientific, Inc.), according to the manufacturer's instructions and were then cultured for 48 h prior being used in subsequent experiments. The control groups were treated with scrambled siRNA. Furthermore, HN-4 cells transfected or not with TRPP2-specific siRNA were cultured into well plates and treated with an AMPK inhibitor (compound C; 20 *μ*M) or activator (AICAR; 1 m*Μ*; both from Sigma-Aldrich; Merck KGaA) to investigate the effects on the AMPK signaling pathways and cell proliferation.

### 2.2. Western Blot Analysis

Western blot assays were performed as previously described [11]. Briefly, total proteins containing the target proteins TRPP2, AMPK, p-AMPK, ACC, p-ACC, PERK, p-PERK, eIF2*α*, and p-eIF2*α* were extracted from HN-4 cells with a detergent extraction buffer. Total protein extracts (30 *μ*g) were loaded into each lane of a 10% sodium dodecyl sulfate polyacrylamide gel, separated by gel electrophoresis, and then transferred onto a polyvinylidene difluoride membrane (EMD Millipore). Following transfer, membranes containing the target proteins were incubated in Tris-buffered saline solution supplemented with 10% nonfat milk for 1 h at room temperature to block nonspecific binding sites. Subsequently, for immunoblots, membranes were first incubated with the primary antibodies against TRPP2 (cat. no. sc-25749), PERK (cat. no. sc-13073), p-PERK (cat. no. sc-32577), eIF2*α* (cat. no. sc-11386), p-eIF2*α* (cat. no. sc-101670; all from Santa Cruz Biotechnology, Inc.), AMPK*α* (cat. no. 2532S), p-AMPK (Thr172 in the *α* subunit) (cat. no. 40H9), ACC (cat. no. C83B10), and p-ACC (Ser79) (cat. no. D7D11; all from Cell Signaling Technology, Inc.) at a 1 : 200 dilution overnight at 4°C and then with the corresponding secondary antibodies for 1 h at room temperature. The immunosignals were detected using an enhanced chemiluminescence detection system (Thermo Fisher Scientific, Inc.). The optical density (OD) of each protein band was analyzed using the ImageJ software (National Institutes of Health) by calculating the integrated OD values. The results are expressed as the relative OD. All assays were performed in triplicate.

### 2.3. Cell Proliferation Assay

Cell proliferation rate was measured using a Cell Counting Kit-8 (CCK-8; Santa Cruz Biotechnology, Inc.). Briefly, HN-4 cells were seeded onto 96-well plates and treated with AICAR or compound C or TRPP2-specific siRNA or in combination. Following incubation at 37°C in 5% CO_2_ for 48 h, each well was supplemented with 10 *μ*l CCK-8 reagent and incubated for 2 h, and subsequently, the absorbance of each well was recorded at a wavelength of 450 nm to determine the cell proliferation rate by calculating the OD value. A total of five independent experiments were conducted.

### 2.4. Statistical Analysis

The SigmaPlot software (version, 16.5) was used to analyze all data. Data are expressed as the mean ± standarderrorofthemean (SEM). Two-tailed, unpaired Student's *t*-test was performed to compare the results between groups, and *P* < 0.05 was considered to indicate a statistically significant difference.

## 3. Results

### 3.1. Changes on the AMPK/ACC and AMPK/PERK/eIF2*α* Pathways in HN-4 Cells

**Figure 1:**
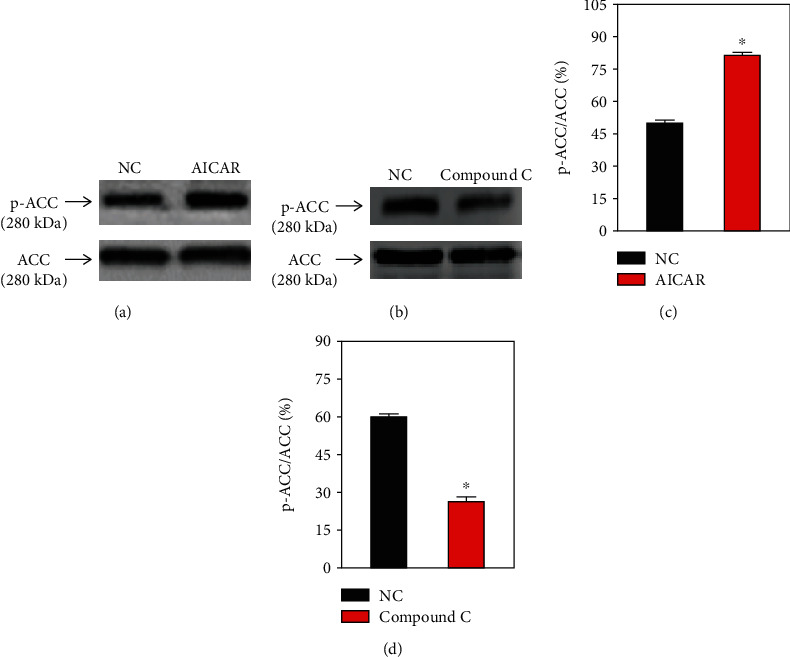
Changes of the AMPK/ACC signaling pathway in HN-4 cells. (a, b) Representative western blot images and (c, d) summary data showing ACC and p-ACC expression in HN-4 cells treated with (a, c) AICAR or (b, d) compound C for 36 h. Values are presented as the means ± SEM (*n* = 3). ^∗^*P* < 0.05. AMPK: AMP-activated protein kinase; ACC: acetyl-CoA carboxylase; p-ACC: phosphorylated ACC; AICAR: 5-aminoimidazole-4-carboxamide-1-*β*-4-ribofuranoside; NC: no treatment (control); SEM: standard error of the mean.

**Figure 2:**
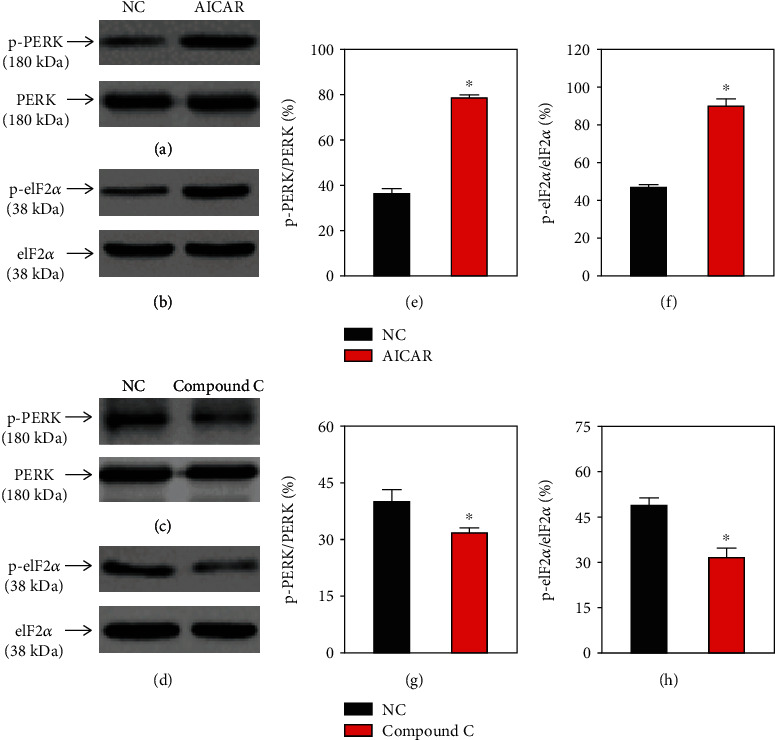
Changes of the PERK/eIF2*α* signaling pathway in HN-4 cells. (a, b) Representative western blot images and (e, f) summary data showing (a) PERK, (e) p-PERK, (b) eIF2*α*, and (f) p-eIF2*α* expression levels in HN-4 cells treated with AICAR. (c, d) Representative western blot images and (g, h) summary data showing (c) PERK, (g) p-PERK, (d) eIF2*α*, and (h) p-eIF2*α* expression levels in HN-4 cells treated with compound C. Values are presented as the means ± SEM (*n* = 3). ^∗^*P* < 0.05. PERK: protein kinase RNA-like endoplasmic reticulum kinase; eIF2*α*: eukaryotic initiation factor 2*α*; p-PERK: phosphorylated PERK; AICAR: 5-aminoimidazole-4-carboxamide-1-*β*-4-ribofuranoside; NC: no treatment (control); SEM: standard error of the mean.

To establish the similar effects of the AMPK/ACC and AMPK/PERK/eIF2*α* signaling pathways in HN-4 cells, western blot analyses were performed to assess the expression levels of ACC, p-ACC, PERK, p-PERK, eIF2*α*, and p-eIF2*α* in the presence of an AMPK activator or inhibitor (*n* = 3). The relative ex levels of p-ACC, p-PERK, and p-eIF2*α* in HN-4 (Figures 1 and 2) were significantly increased following treatment with an AMPK activator (AICAR) and significantly decreased in cells treated with an AMPK inhibitor (compound C).

### 3.2. Knockdown of TRPP2 Suppresses the PERK/eIF2 Pathway and Upregulates the AMPK/ACC Pathway

**Figure 3:**
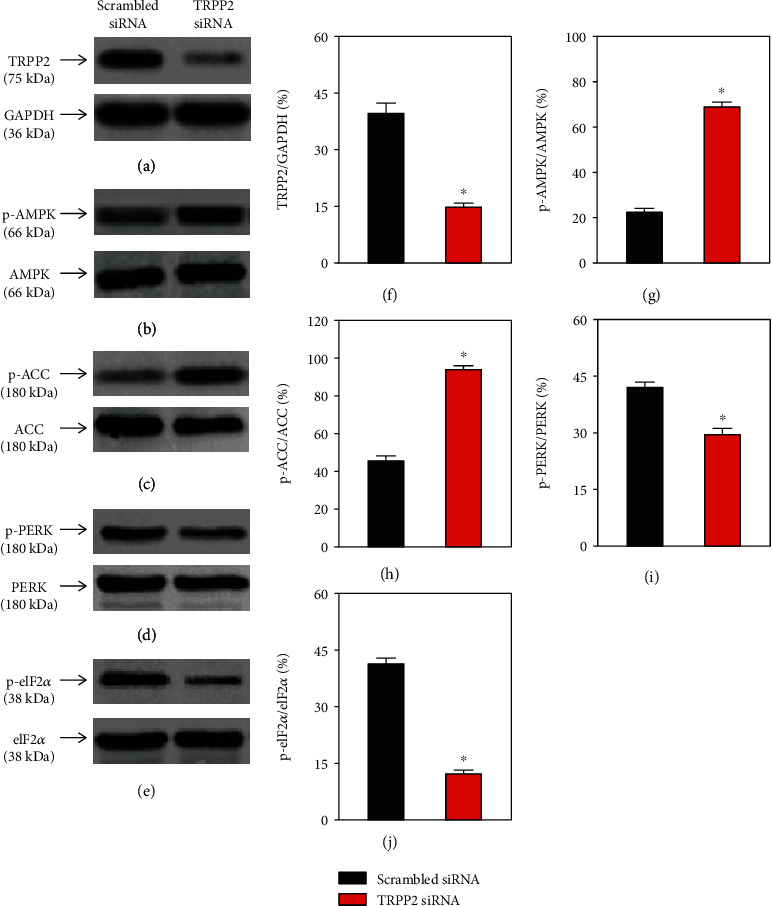
Knockdown of TRPP2 inhibited the PERK/eIF2*α* and activated the AMPK/ACC pathway in HN-4 cells. (a–e) Representative western blot images and (f–j) summary data showing the expression of (a, f) TRPP2, (b) AMPK, (g) p-AMPK, (c) ACC, (h) p-ACC, (d) PERK, (i) p-PERK, (e) eIF2*α*, and (j) p-eIF2*α* in HN-4 cells transfected with scrambled siRNA or TRPP2-specific siRNA. Values are presented as the means ± SEM (*n* = 3). ^∗^*P* < 0.05. TRPP2: transient receptor potential polycystic 2; PERK: protein kinase RNA-like endoplasmic reticulum kinase; eIF2*α*: eukaryotic initiation factor 2*α*; AMPK: AMP-activated protein kinase; ACC: acetyl-CoA carboxylase; p-AMPK: phosphorylated AMPK; PERK: protein kinase RNA-like endoplasmic reticulum kinase; eIF2*α*: eukaryotic initiation factor 2*α*; SEM: standard error of the mean.

Whether the effect of TRPP2 on cell proliferation is associated with the AMPK signaling pathways remains unknown. To reveal the underlying mechanisms of TRPP2, western blot analyses were performed to determine the relative expression levels of p-AMPK, p-ACC, p-PERK, and p-eIF2*α*. TRPP2-specific siRNA increased the expression levels of p-AMPK and p-ACC and decreased those of p-PERK and p-eIF2*α* (*n* = 3), indicating that silencing of TRPP2 downregulated the PERK/eIF2*α* signaling pathway in HN-4 (Figure 3). The above results were consistent with a previous study, suggesting that TRPP2 negatively regulated cell proliferation via upregulating the PERK/eIF2*α* signaling pathway [26]. Despite the fact that the association between TRPP2 and AMPK is poorly understood, our results demonstrating the activation of AMPK/ACC signaling pathway in HN-4 cells transfected with TRPP2-specific siRNA are a novel finding that deserves further investigation.

### 3.3. Inhibition or Activation of the AMPK/ACC Signaling Pathway Further Enhances or Inhibits TRPP2-Specific siRNA-Mediated HN-4 Cell Proliferation

**Figure 4:**
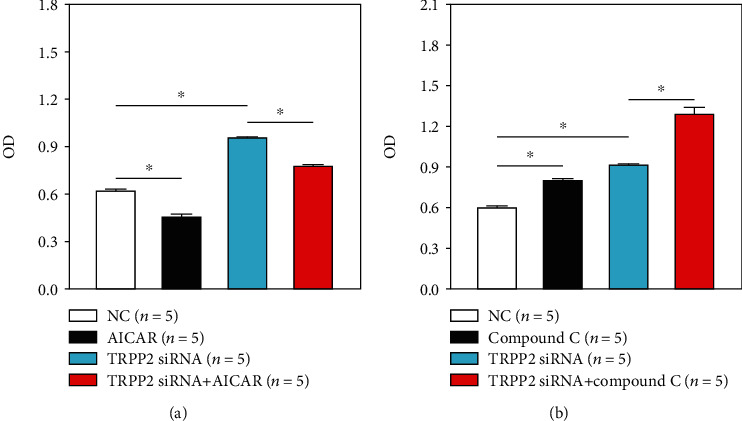
Effect of blockade or stimulation of the AMPK/ACC signaling pathway on the proliferation of HN-4 cells treated or not with si-TRPP2. Summary data showing the proliferation rate of HN-4 cells transfected with scrambled siRNA or TRPP2-specific siRNA and treated with AICAR or compound C for 36 h (expressed as OD). Values are presented as the means ± SEM (*n* = 5). ^∗^*P* < 0.05. AMPK: AMP-activated protein kinase; ACC: acetyl-CoA carboxylase; TRPP2: transient receptor potential polycystic 2; AICAR: 5-aminoimidazole-4-carboxamide-1-*β*-4-ribofuranoside; NC: no treatment (control); OD: optical density; SEM: standard error of the mean.

In the present study, a TRPP2-specific siRNA was used as a tool to investigate the effects of TRPP2 silencing on HN-4 cell proliferation. As shown in Figure 4, compared with the control group, treatment of cells with AICAR (Figure 4(a)) or compound C (Figure 4(b)) reduced or enhanced cell proliferation, respectively. In addition, following cell transfection with TRPP2-specific siRNA, the HN-4 cell proliferation rate was significantly increased. Furthermore, in the TRPP2-specific siRNA or scrambled siRNA groups, treatment of cells with compound C or AICAR further accelerated or reduced HN-4 cell proliferation, respectively (Figures 4(a) and 4(b)) (*n* = 5).

## 4. Discussion

**Figure 5:**
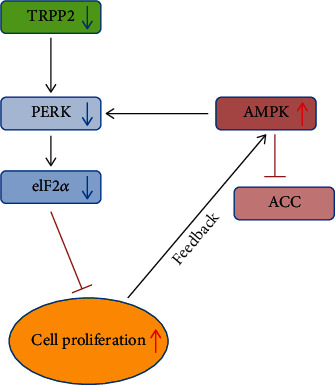
Interactions among the TRPP2/PERK/eIF2*α*, AMPK/ACC, and AMPK/PERK/eIF2*α* signaling pathways. Activation of the AMPK/ACC and AMPK/PERK/eIF2*α* signaling pathways inhibited HN-4 cell proliferation. Knockdown of TRPP2 promoted HN-4 cell proliferation via downregulating the PERK/eIF2*α* signaling pathway. The AMPK/ACC signaling pathway was activated to inhibit cell growth in response to increased cell proliferation via a feedback mechanism. TRPP2: transient receptor potential polycystic 2; PERK: protein kinase RNA-like endoplasmic reticulum kinase; eIF2*α*: eukaryotic initiation factor 2*α*; AMPK: AMP-activated protein kinase; ACC: acetyl-CoA carboxylase.

The present study examined whether knockdown of TRPP2 affected HN-4 cell proliferation and, if so, the mechanism supporting this effect. The major findings of the present study were as follows: (i) the effects of AMPK/ACC and AMPK/PERK/eIF2*α* signaling pathways were established in HN-4 cells and were consistent with those reported in other cell lines; (ii) knockdown of TRPP2 increased the relative expression levels of p-AMPK and p-ACC and decreased those of p-PERK and p-eIF2*α*; (iii) knockdown of TRPP2 increased HN-4 cell proliferation, which was further increased in cells treated with an AMPK inhibitor and inhibited in those treated with an AMPK agonist. Taken together, these results indicated that knockdown of TRPP2 promoted HN-4 cell proliferation via inhibiting the PERK/eIF2*α* signaling pathway, while the AMPK/ACC signaling pathway was activated by a feedback mechanism in response to the increased cell proliferation rate. Therefore, these findings provided experimental evidence furthering our understanding of TRPP2-specific siRNA-mediated HN-4 cell proliferation and clarifying the associations between AMPK/ACC, AMPK/PERK/eIF2*α*, and TRPP2/PERK/eIF2*α* signaling pathways in cell proliferation (Figure 5). Furthermore, these results importantly revealed potential targets for the treatment of diseases associated with mutant or low expression levels of TRPP2.

Immunotherapy and gene therapy have been suggested to be successful treatment strategies for HNC. For example, a clinical trial in 2016 showed that the anti-PD-1 antibodies, nivolumab and pembrolizumab, exhibited great efficacy in the treatment of recurrent and metastatic head and neck squamous cell carcinoma [7]. Gene therapy of this cancer type is vast and includes several treatment mechanisms such as targeting oncogenes, gene corrective therapy, and cytoreductive and immunomodulatory strategies [8]. However, the response rate remains low in some patients. Therefore, identifying novel specific therapeutic targets for HNC is urgent.

AMPK is a kinase that phosphorylates ACC and regulates cell energy metabolism. It has been reported that activation of the AMPK signaling pathways inhibits proliferation of retinoblastoma and prostate cancer cells [16, 17]. Oxyphenisatin acetate exerts antiproliferative activity in breast cancer cells via activating the PERK/eIF2*α* and AMPK/mTOR signaling pathways, while it has been reported that orlistat inhibits endometrial cancer cell proliferation via attenuating fatty acid metabolism, activating the AMPK/mTOR signaling pathway and inducing cellular stress mediated by the increased PERK expression [13, 23, 24]. These findings indicate that activation of the PERK/eIF2*α* and AMPK/mTOR signaling pathways inhibits cancer cell proliferation. In addition, several studies have shown that AMPK exerts an antilipolytic effect via activating the PERK/eIF2 signaling pathway in TNF-*α*-induced adipocytes, while AMPK activation by astragaloside IV decreases free fatty acid-induced ER stress [22, 25]. Therefore, as the activation of AMPK/ACC, AMPK/mTOR, and AMPK/PERK/eIF2*α* signaling pathways reduces cell proliferation, investigating drug therapies targeting the AMPK signaling pathways for the treatment of HNC is important. Furthermore, the present study demonstrated that the p-ACC, p-PERK, and p-eIF2*α* relative expression levels were increased or decreased in HN-4 cells following treatment with an AMPK activator or inhibitor, respectively. Additionally, treatment with an AMPK activator decreased HN-4 cell proliferation, which was increased following treatment with an AMPK inhibitor. The aforementioned results confirmed that activation of the AMPK/ACC and AMPK/PERK/eIF2*α* signaling pathways inhibited HN-4 cell proliferation, while their blockade had the opposite effects.

TRPP2 has been shown to attenuate Madin-Darby Canine Kidney (MDCK) and HEK293T cell proliferation via activating the PERK/eIF2*α* signaling pathway, thus suggesting that TRPP2/PERK/eIF2*α* signaling pathway is involved in cell proliferation [26]. Furthermore, TNF-*α*-mediated suppression of TRPP2 accelerates Hep-2 cell proliferation via downregulating the PERK/eIF2*α* signaling pathway [11]. On the basis of these findings, the present study hypothesized that the AMPK signaling pathways were involved in TRPP2-mediated cell proliferation. The results demonstrated that the relative expression levels of p-AMPK and p-ACC were increased, while those of p-PERK and p-eIF2*α* were decreased in TRPP2-silenced HN-4 cells. Previous studies have indicated that the increased p-eIF2*α* expression is associated with reduced global protein synthesis, including the cell-cycle factor cyclin D1, resulting in cell growth arrest [19, 27, 28]. Therefore, in the present study, TRPP2-mediated cell proliferation was promoted via downregulating the PERK/eIF2*α* signaling pathway. Although this finding is consistent with a previous study, the understanding of the upregulation mechanism of the AMPK/ACC signaling pathway is challenging [26].

Tumor suppressor LKB1 and Ca^2+^/calmodulin-dependent protein kinase kinase *β* (CaMKK*β*) are two upstream kinases of AMPK. The LKB1-mediated phosphorylation of AMPK*α* at Thr172 residue is enhanced by AMP binding to the AMPK*γ* subunit, whereas CaMKK*β* activates AMPK by elevating the intracellular Ca^2+^ concentration [29, 30]. A previous study has revealed that TRPP2 enhances intracellular Ca^2+^ release and amplifies the Ca^2+^ signal via the Ca^2+^-induced Ca^2+^-release (CICR) mechanism, thus contributing to disease development [31]. However, the direct interaction between TRPP2 and AMPK has not yet been demonstrated. Furthermore, it has been suggested that knockdown of TRPP2 inhibits inositol trisphosphate and ATP-induced Ca^2+^ release [11, 32]. Therefore, these findings indicated that the activation of AMPK in the presence of TRPP2-specific siRNA was not mediated by physical interaction or the Ca^2+^ signal. As AMPK activation inhibited cell growth, the present study considered that the TRPP2-specific siRNA-mediated activation of AMPK/ACC signaling pathway was indirect and was possibly enhanced by increased HN-4 cell proliferation [33]. Consistent with this hypothesis, the results of the present study demonstrated that knockdown of TRPP2 enhanced HN-4 cell proliferation, which was further increased following treatment with an AMPK inhibitor and decreased in AMPK activator-treated HN-4 cells. Therefore, activation of the AMPK/ACC signaling pathway was mediated by a feedback in response to increased cell proliferation rate, while p-AMPK and p-ACC upregulation was a compensatory alteration. Taken together, it was a plausible conclusion that knockdown of TRPP2 promoted HN-4 cell proliferation via downregulating the PERK/eIF2*α* signaling pathway, and the AMPK/ACC signaling pathway was, in turn, activated in response to increase cell proliferation by a feedback mechanism. These findings provided deeper insights into the interactions among the TRPP2/PERK/eIF2*α*, AMPK/ACC, and AMPK/PERK/eIF2*α* signaling pathways. In our previous study, we found that increased TRPP2 promoted the invasion and metastasis of Hep2 cell, while TRPP2 siRNA markedly suppressed ATP-induced Ca^2+^ release, wound healing, and cell invasion in Hep2 cell [34]. The different roles of TRPP2 in cell behaviors were similar to the effect of integrin *α*2*β*1 on prostate cancer cell that integrin *α*2*β*1 decelerated cell proliferation and promoted survival and invasion of prostate cancer cells [35]. Besides, TRPP2 was found to be involved in regulating cell apoptosis [12], and TRPP2 siRNA enhanced the growth of MDCK cells and cyst formation [36], which suggested that TRPP2 participates in different biological processes and exerts different biological behaviors, which needs further study to explore the mechanisms underpinning its effects. Consistent with previous studies suggesting that AMPK activation is applicable for managing ADPKD and reducing renal cystogenesis, the current findings show that activation of the AMPK signaling pathways may be effective to delay tumor growth and renal cystogenesis with the low TRPP2 expression [37, 38].

Altogether, the increases in p-PERK, p-eIF2*α*, and cell proliferation in our study kept consistent with the findings of Liang et al. that increased or decreased TRPP2 level greatly downregulated or upregulated the proliferation of MDCK and/or HEK293T cells via the PERK/eIF2*α* signaling pathway, respectively [26]. Moreover, Brewer et al. demonstrated that protein synthesis including the cell-cycle factors such as cyclin D1 was repressed in response to elevated p-eIF2*α* level, thus leading to the inhibition of cell proliferation [19, 27, 28]. We could conclude that the inhibition of the TRPP2/PERK/eIF2*α* signaling pathway was related to increased cell growth. In addition, AMPK was mainly activated by tumor suppressor LKB1, and CaMKK*β* was determined to trigger AMPK activation via elevating cell Ca^2+^ [39]. Therefore, we firstly speculated that TRPP2-mediated AMPK activation may be achieved by the two mechanisms mentioned above. However, we further evaluated that the direct interaction between TRPP2 and AMPK has not been demonstrated, and TRPP2 could promote intracellular Ca^2+^ release and amplify the intracellular Ca^2+^ signal [31], and knockdown of TRPP2 significantly decreased ATP-induced Ca^2+^ release [11]. Thus, we realized that TRPP2-mediated AMPK activation may be through an indirect mechanism. In combination with the fact that AMPK activation suppressed cell proliferation [33], we concluded that the activation of AMPK in the presence of TRPP2-specific siRNA was neither through the physical interaction nor through the Ca^2+^ signal, and AMPK was possibly activated by feedback mechanism in response to increased cell growth.

In the present study, the limitations lie in that the activation of AMPK by the feedback mechanism in response to TRPP2 siRNA was not a proven hypothesis, which needs further studies to demonstrate it. In this study, we observed that increased cell proliferation was mediated by the TRPP2/PERK/eIF2*α* signaling pathway in the presence of TRPP2 siRNA. Because no direct evidence suggested that there existed direct interaction between TRPP2 and AMPK, we proposed that elevated AMPK level was mediated by the increased cell proliferation. However, the mechanisms underlying the hypothesis should be further demonstrated in future.

The present study demonstrated that knockdown of TRPP2 increased HN-4 cell proliferation via downregulating the PERK/eIF2*α* signaling pathway and that the AMPK/ACC signaling pathway was possibly activated by a feedback mechanism. Furthermore, the results suggested that the activation of AMPK signaling pathway may be applied for the treatment of mutant TRPP2-related diseases, such as ADPKD, and conditions associated with TRPP2 downregulation, including tumors.

## Data Availability

The datasets used and/or analyzed during the current study are available from the corresponding author on reasonable request.

## Abbreviations

ACC: Acetyl-CoA carboxylase
ADPKD: Autosomal dominant polycystic kidney disease
AICAR: 5-Aminoimidazole-4-carboxamide-1-*β*-4-ribofuranoside
AMPK: AMP-activated protein kinase
Ca^2+^: Calcium
CaMKK*β*: Calmodulin-dependent protein kinase kinase *β*
CCK-8: Cell counting Kit-8
eIF2*α*: Eukaryotic initiation factor 2*α*
ER: Endoplasmic reticulum
HNC: Head and neck cancer
mTOR: The mammalian target of rapamycin
ODs: Optical densities
p-ACC: Phosphorylated ACC
PERK: Protein kinase RNA-like endoplasmic reticulum kinase
siRNA: Small interfering RNA
TNF-*α*: Tumor necrosis factor-*α*
TRPP2: Transient receptor potential polycystic 2.
